# Role of JNK Activation and Mitochondrial Bax Translocation in Allicin-Induced Apoptosis in Human Ovarian Cancer SKOV3 Cells

**DOI:** 10.1155/2014/378684

**Published:** 2014-07-08

**Authors:** Ling Xu, Jin Yu, Dongxia Zhai, Danying Zhang, Wei Shen, Lingling Bai, Zailong Cai, Chaoqin Yu

**Affiliations:** ^1^Department of Traditional Chinese Gynecology, Changhai Hospital, Second Military Medical University, Shanghai 200433, China; ^2^Traditional Chinese Medicine University of Shanghai, Shanghai 201203, China; ^3^Department of Biochemistry and Molecular Biology, Second Military Medical University, Shanghai 200433, China

## Abstract

*Background*. Allicin, the major component of freshly crushed garlic, is one of the most biologically active compounds of garlic; it has been reported to induce apoptosis in cancer cells; however, the mechanism by which allicin exerts its apoptotic effects is not fully understood. The aim of the present study was to further elucidate the apoptotic pathways induced by allicin in the human ovarian cancer cell line SKOV3. *Methods*. Cell proliferation and apoptosis were measured by cell-counting assay and flow cytometry analysis. Activation of the signaling pathway was screened by human phospho-kinase array analysis, and the activated pathway and its related proteins were further confirmed by western blot analysis. *Results*. Allicin induced SKOV3 cell apoptosis and JNK phosphorylation in a time- and dose-dependent manner, but these were significantly blocked by SP600125 (an inhibitor of JNK). The findings suggest that JNK phosphorylation is related to the action of allicin on SKOV3 cells. Furthermore, JNK activation induced Bcl-2 family activation, triggered mitochondria-mediated signaling pathways, and led to the translocation of a considerable amount of Bax and cytochrome *c* release. *Conclusions*. JNK activation and mitochondrial Bax translocation are involved in allicin-induced apoptosis in SKOV3 cells. Our data input new insights to the literature of allicin-induced apoptosis.

## 1. Introduction

Ovarian cancer is a leading cause of cancer-related death in western countries, and its incidence in Asia is increasing. At present, cytoreductive surgery and chemotherapy were considered to be the conventional treatments of ovarian cancer. However, the success rate of surgery is only 35~50% and the multiple drug resistance (MDR) caused by chemotherapy results in the 5-year-survival rate of advanced ovarian cancer patients in only 20~30%. Therefore, although ovarian cancer is a well-studied cancer, progress in its prevention or cure is still needed.

Garlic (*Allium sativum*) has long been used as food and remedy in oriental countries. Researches have shown that garlic possesses a variety of biological activities, including antiatherosclerotic, antihypertensive, antimicrobial, anticancer, immunomodulatory, radioprotective, and potential antiaging effects [1, 2]. Allicin, the major component of freshly crushed garlic, is one of the most biologically active compounds of garlic [3] and is formed from alliin via the action of alliinase [4, 5]. Allicin has obvious inhibitory effects on different kinds of tumor cells such as gastric cancer, colon cancer, liver cancer, and lung cancer and has been put into clinical treatment as an aid cancer drug.

Activation of apoptosis signaling pathways may be responsible for treatment of malignant diseases. Two apoptotic pathways that converge on caspase-3, with one involving caspase-8 and the other involving the mitochondrial release of cytochrome *c* as well as the activation of caspase-9, have been described [6, 7]. Mitochondrial apoptosis signaling is initiated by changes in mitochondrial membrane integrity. Soluble apoptosis signaling molecules, such as cytochrome *c*, localized in the mitochondrial intermembrane space are released into the cytosol upon apoptosis induction [8]. Released cytochrome *c* associates with Apaf-1 [9, 10] and activates procaspase-9 in a multimeric complex, the apoptosome [11–13]. Active caspase-9 in turn processes the downstream effector caspases 3, 6, and 7 [14]. Thus, the release of cytochrome *c* into the cytosol represents a pivotal step of apoptosis signaling and analysis of mitochondrial cytochrome *c* release might therefore identify apoptosis in mitochondrial signaling [15]. Studies have demonstrated that some Bcl-2 family members (e.g., Bax, Bcl-xL, Mcl-1, Bcl-2, and Bid) located in the mitochondrial membrane can alter the permeability of the membrane and trigger the activation of caspases [16], thereby, leading to apoptotic cell death. Allicin has been reported to induce apoptosis in human epithelial carcinoma cells through the mitochondrial release of apoptosis induce factor (AIF) and protein kinase A was found to play an important role in caspase-independent apoptotic pathways [17].

The proapoptotic effects of allicin against cancer cells were provided by in vitro studies [18]; however, the mechanism by which allicin exerts its apoptotic effects especially on ovarian cancer is not fully understood. The present study offers new evidence showing that activation of JNK and mitochondrial translocation of Bax are involved in allicin-induced apoptosis in human ovarian cancer SKOV3 cells.

## 2. Materials and Methods

### 2.1. Materials

Allicin was purchased from Shanghai Harvest Pharmaceutical Co., Ltd. (Shanghai, China). The purity of allicin used in the experiments was ≥90%, as determined by HPLC. Mouse anti-Hsp60 monoclonal antibody and anti-Bax monoclonal antibody 2D2 were purchased from Santa Cruz Biotechnology, Inc (USA). Antibodies against cytochrome *c* and the JNK inhibitor SP600125 were obtained from Beyotime Institute of Biotechnology, whereas antibodies against *β*-actin, phospho-JNK, and JNK were purchased from Cell Signaling Technology. Human Phospho-Kinase Array (catalog number ARY003) was obtained from R&D Systems Co. Ltd. (USA). RPMI-1640 medium and fetal bovine serum were purchased from GIBCO (USA).

### 2.2. Cell Culture and Treatment

The human ovarian cell line SKOV3 was obtained from the China Center for Type Culture Collection (Wuhan, China). The cells were routinely cultured in RPMI-1640 medium supplemented with 10% fetal bovine serum in a humidified atmosphere with 5% CO_2_ incubation at 37°C. Treatments were performed with different amounts of allicin, ranging from 0 to 100 *μ*g/mL. Unless otherwise specified, the concentration of allicin selected for all the experiments was 25 *μ*g/mL; an equal amount of phosphate buffered saline or dimethyl sulfoxide was added to cells as control.

### 2.3. Cell Proliferation and Apoptosis Assay

SKOV3 cells (2 × 10^4^) were seeded in each well of 96-well plates and incubated at various concentrations of allicin for different periods. After treatment, the proliferative potential of the cells was analyzed using Cell Counting Kit-8 (Dojindo, Kumamoto, Japan) according to the manufacturer's protocol. For apoptosis assay, the SKOV3 cells were grown to approximately 75% confluence in 6-well plates and then treated with or without allicin (25 *μ*g/mL, 48 h) and/or JNK inhibitors (20 *μ*M, 30 min). After treatment, the cells were collected, washed, and resuspended in 200 *μ*L of binding buffer at 2 × 10^5^ cells/mL. The samples were subsequently incubated with 2.5 *μ*L of Annexin V-FITC and 5 *μ*L of propidium iodide in the dark for 15 min at room temperature and then analyzed by flow cytometry (Miltenyi, Germany).

### 2.4. Phospho-Kinase Proteome Profiling and Western Blot Analysis

The cells were seeded at a density of 1 × 10^7^ cells per 60 cm^2^ dish, cultured for 24 h, treated using indicated concentrations of allicin for 48 h, and processed using Human Phospho-Kinase Array Kit (Proteome Profiler; R&D Systems, Minneapolis, USA) following the manufacturer's instructions. Phospho-kinase array data were developed on X-ray films following exposure to chemiluminescent reagents. The results were confirmed by western blot analysis, as previously described [19].

### 2.5. Detection of Bax Translocation and Cytochrome *c* Release in Mitochondria

Crude mitochondrial and cytosolic extracts were prepared from SKOV3 cells with indicated treatments, as described by Parone et al. [20]. Bax and cytochrome *c* in the cytosol and mitochondria were detected by western blot analysis.

### 2.6. Statistical Analysis

Data were obtained from three independent experiments and expressed as mean ± SD. Differences were analyzed using Student's *t*-test or one-way ANOVA, as appropriate. *P* < 0.05 was considered statistically significant.

## 3. Results

### 3.1. Allicin Inhibits SKOV3 Cell Proliferation and Induces Apoptosis

**Figure 1:**
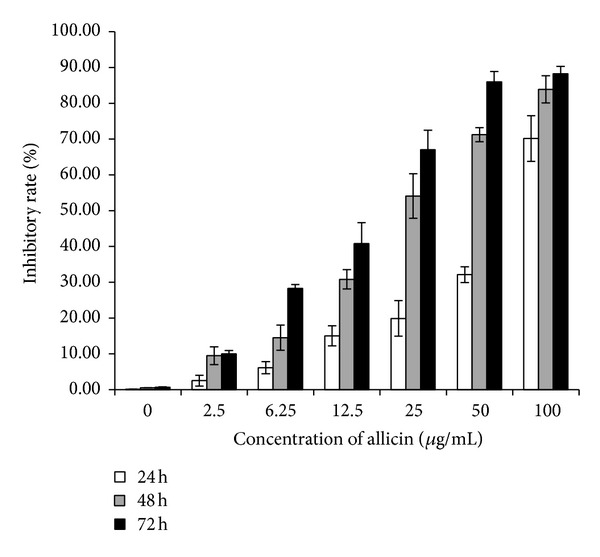
Inhibitory effect of allicin on SKOV3 cell proliferation. SKOV3 cells were treated with various doses of allicin for 24, 48, and 72 h. Cell proliferation was determined using cell-counting assay and expressed as the percentage of the absorbance value obtained without allicin.

**Figure 2:**
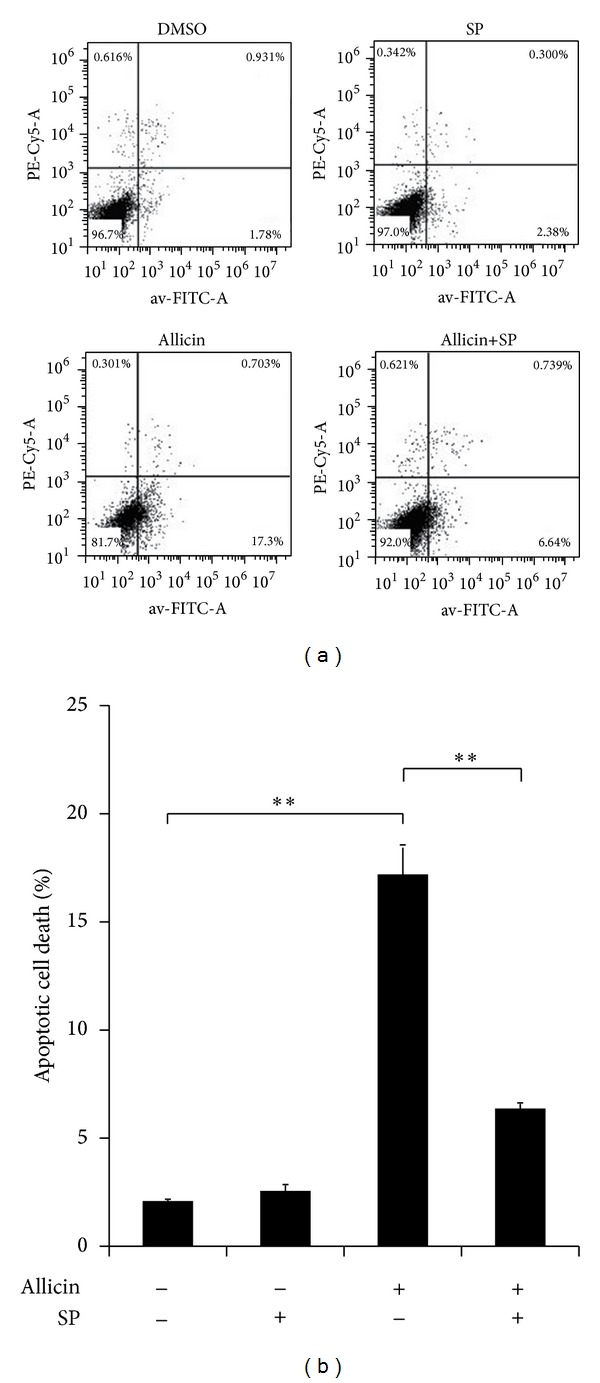
Flow cytometry analysis of allicin and/or SP600125 in SKOV3 cell apoptosis. SKOV3 cells were pretreated with 20 *μ*M SP600125 for 30 min before incubation with 25 *μ*g/mL of allicin, and apoptotic cells were measured by cytometry after 48 h. Data (mean ± SD) are representative of three experiments. (a) is a representative figure and (b) is a statistical graph. Asterisks indicate statistically significant difference (**P < 0.01).

The antiproliferative effect of allicin on SKOV3 cells was examined by exposing the cells to different concentrations of allicin for 24, 48, and 72 h. Cell growth was inhibited in a dose- and time-dependent manner (Figure 1). In the presence of 25 *μ*g/mL of allicin, SKOV3 cells exhibited approximately 60% inhibition of proliferation after treatment for 48 h. As such, this concentration and the treatment time were used in the following experiments. Flow cytometry analysis showed that allicin induced apoptosis significantly, which was also significantly blocked by pretreatment with SP600125 (Figure 2); however, SP600125 alone could not inhibit apoptosis.

### 3.2. Activation of the Signaling Pathway by Allicin in SKOV3 Cells

**Figure 3:**
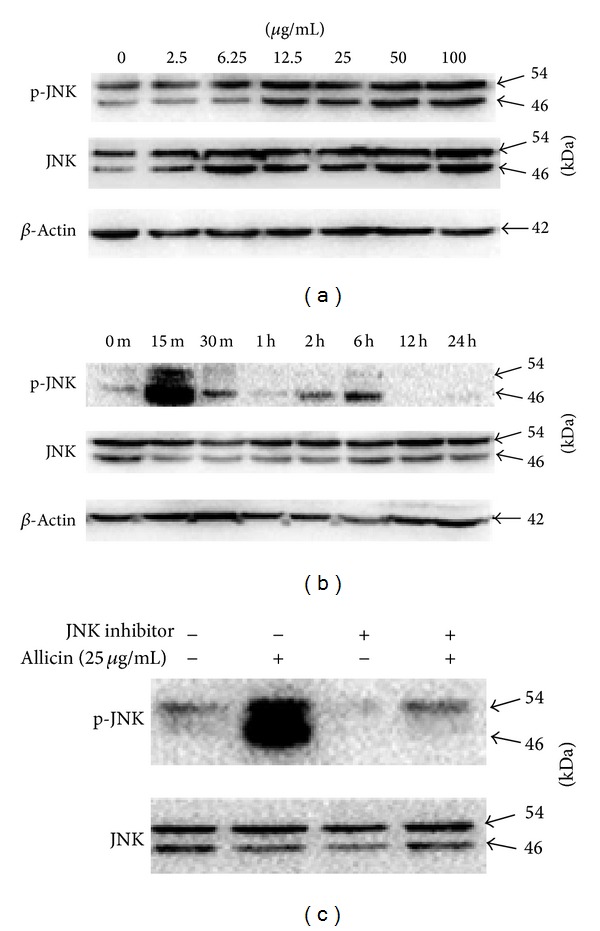
Effect of allicin and/or SP600125 on the phosphorylation of JNK in SKOV3 cells. (a) Treatment with various concentrations of allicin for 15 min. (b) Treatment with 25 *μ*g/mL of allicin at indicated times. (c) Pretreatment with 20 *μ*M SP600125 for 30 min before incubation with 25 *μ*g/mL of allicin for 15 min; JNK phosphorylation was measured by western blot analysis after 48 h.

Human phospho-kinase array assays were performed to discover which signaling pathways are involved in allicin-induced SKOV3 cell apoptosis. The AKT and JNK pathways were activated (see supplementary data in Supplementary Material available online at http://dx.doi.org/10.1155/2014/378684). As activation of the JNK pathway is a novel finding in this setting, we focused on it in the following experiments. Phospho-JNK increased in a dose-dependent manner (Figure 3(a)), and peak phosphorylation was detected at 15 min—when the cells were treated with 25 *μ*g/mL of allicin (Figure 3(b)). Furthermore, SP600125 could partially inhibit JNK phosphorylation as activated by allicin (Figure 3(c)), revealing that allicin-induced apoptosis is related to the JNK MAPK signaling pathway in SKOV3 cells.

### 3.3. JNK Activation by Allicin Results in Bax Translocation and Cytochrome *c* Release in Mitochondria

**Figure 4:**
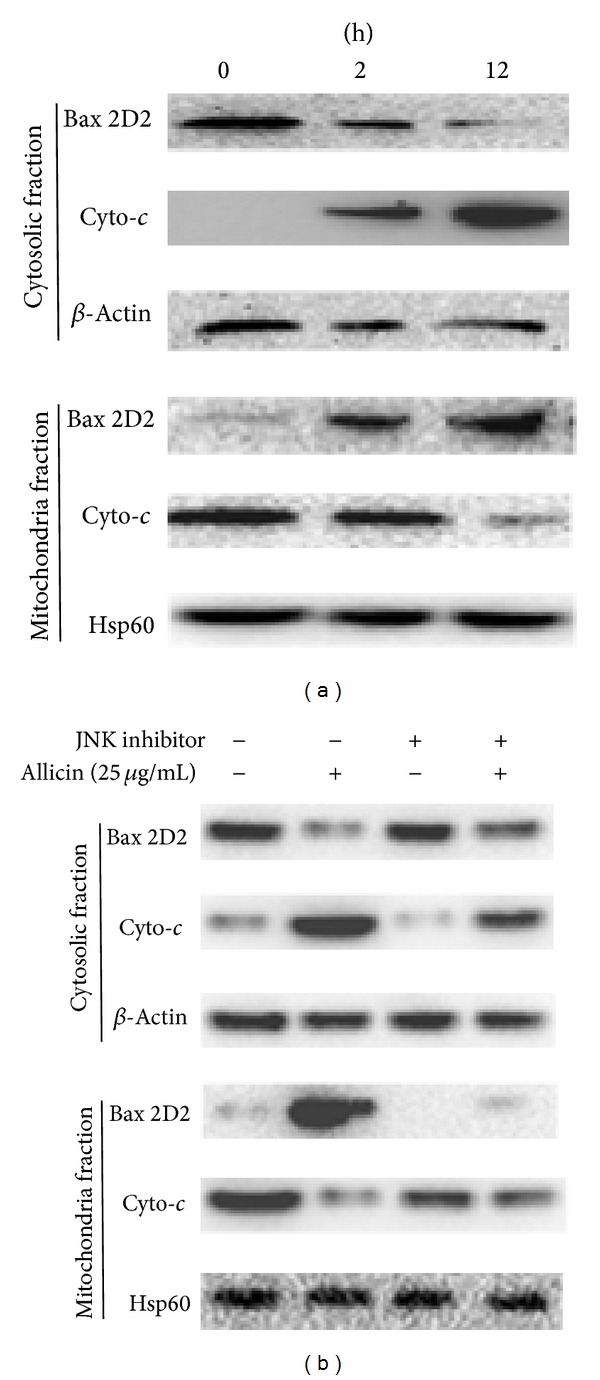
Western blot analysis showing cytochrome *c* and Bax levels in response to allicin. (a) SKOV3 cells were treated with 25 *μ*g/mL of allicin for 12 h. Subsequently, cytosolic and mitochondrial fractions were prepared and western blot analysis was carried out (20 *μ*g of protein) as described in Materials and Methods. (b) Pretreatment with or without the JNK inhibitor SP600125 for 30 min, followed by treatment with allicin for 12 h to analyze Bax and cytochrome *c*. Data are representative of three independent experiments showing a similar pattern of expression. *β*-Actin and Hps60 were used as internal control.

The Bax (2D2) and cytochrome *c* levels in the mitochondrial and cytosolic fractions were examined to further elucidate whether the JNK pathway is involved in downstream molecular events of apoptosis. As shown in Figure 4(a), the mitochondrial Bax level decreased in a time-dependent manner but simultaneously increased in the cytosolic fraction. The opposite was observed for the cytochrome *c* level. Interestingly, SP600125 markedly blocked cytochrome *c* release from mitochondria in SKOV3 cells exposed to allicin (Figure 4(b)). Allicin-induced JNK clearly leads directly to an increase in cytochrome *c* content. These biochemical changes confirm that allicin-induced apoptosis is mediated by JNK activation.

## 4. Discussion

Apoptosis, programmed cell death process, is an important way to remove aging, damage, and mutation of cells. Along with the in-depth study of apoptosis and its mechanisms, researchers come to realize that inducing tumor cell apoptosis is an effective way for the treatment of the tumor [21]. Therefore, exploring new therapy of regulating the cellular mechanisms and inducing apoptosis to treat tumors is becoming one of the hotspot researches in the field of oncology.

Apoptosis is a tightly regulated process controlled by several signaling pathways, such as the caspase and mitochondrial pathways [22]. The Bcl-2 family of proteins, either proapoptotic (Bax) or antiapoptotic (Bcl-2) proteins, plays an important role in apoptosis that leads to the release of cytochrome *c* from mitochondria [23]. Similarly, mitochondria are known to play a central role in mediating “intrinsic death signals” and could therefore serve as a novel target for chemotherapy. Cytochrome *c* is a mitochondrial protein whose release into the cytosol is regulated by Bcl-2 family members [24]. Once it is released into the cytosol, cytochrome *c* interacts with procaspase-9, after which it switches on caspase-3 or caspase-7, leading to apoptosis [25].

Recent research has shown that MAPK proteins are important mediators of apoptosis induced by stressful stimuli [26]. JNK and p38 MAPK are collectively termed “stress-activated protein kinases” because they are activated by various stress-related stimuli and chemotherapy drugs [27]. The JNK signaling pathway has been reported to affect members of the Bcl-2 family. For example, JNK not only can inactivate antiapoptotic Bcl-2 proteins but also can activate the mitochondrial translocation of Bax [28].

In the present study, allicin activated the AKT, P53, and JNK (c-Jun) pathway in SKOV3 cells by human phospho-kinase array analysis; however, the signals were developed weakly, for the control signal was extremely strong (supplementary data). Since the JNK pathway was novel finding in this setting, we focused on the JNK pathway in this study and the JNK activation pattern was further confirmed by western blot. JNK activation subsequently induced mitochondrial Bax translocation and the release of cytochrome *c* from mitochondria into the cytosol. SP600125 could markedly block these actions. In addition, the expression of Bcl-xL slightly decreased following treatment with allicin (data not shown). These results indicate that caspase-independent pathways are involved in allicin-induced apoptosis. In conclusion, our data provide new evidence that allicin can activate the JNK pathway, which leads to mitochondrial Bax translocation and mitochondrial release of cytochrome *c*, thus inducing SKOV3 cell apoptosis.

## Supplementary Material

Supplementary Data. Result of human phospho-kinase array assays.
Allicin activated the AKT, P53, and JNK (c-Jun) pathway in SKOV3 cells were detected by human phospho-kinase array analysis. As shown, the first photo was negative control while the second was allicin (12.5*μ*g/ml) intervention group; As a result, the signals of AKT and P53 were weakly different between the two photos; However, the signal of JNK was extremely increased in allicin group compared with the negative control. Therefore, JNK pathway was novel finding in this setting.
