# Supplementary material for: Role of JNK Activation and Mitochondrial Bax Translocation in Allicin-Induced Apoptosis in Human Ovarian Cancer SKOV3 Cells
Source: Evid Based Complement Alternat Med. 2014 Jul 8;2014:378684. doi: 10.1155/2014/378684 (PMC4109593; doi:10.1155/2014/378684)

Supplementary Data. Result of human phospho-kinase array assays

The editable figure

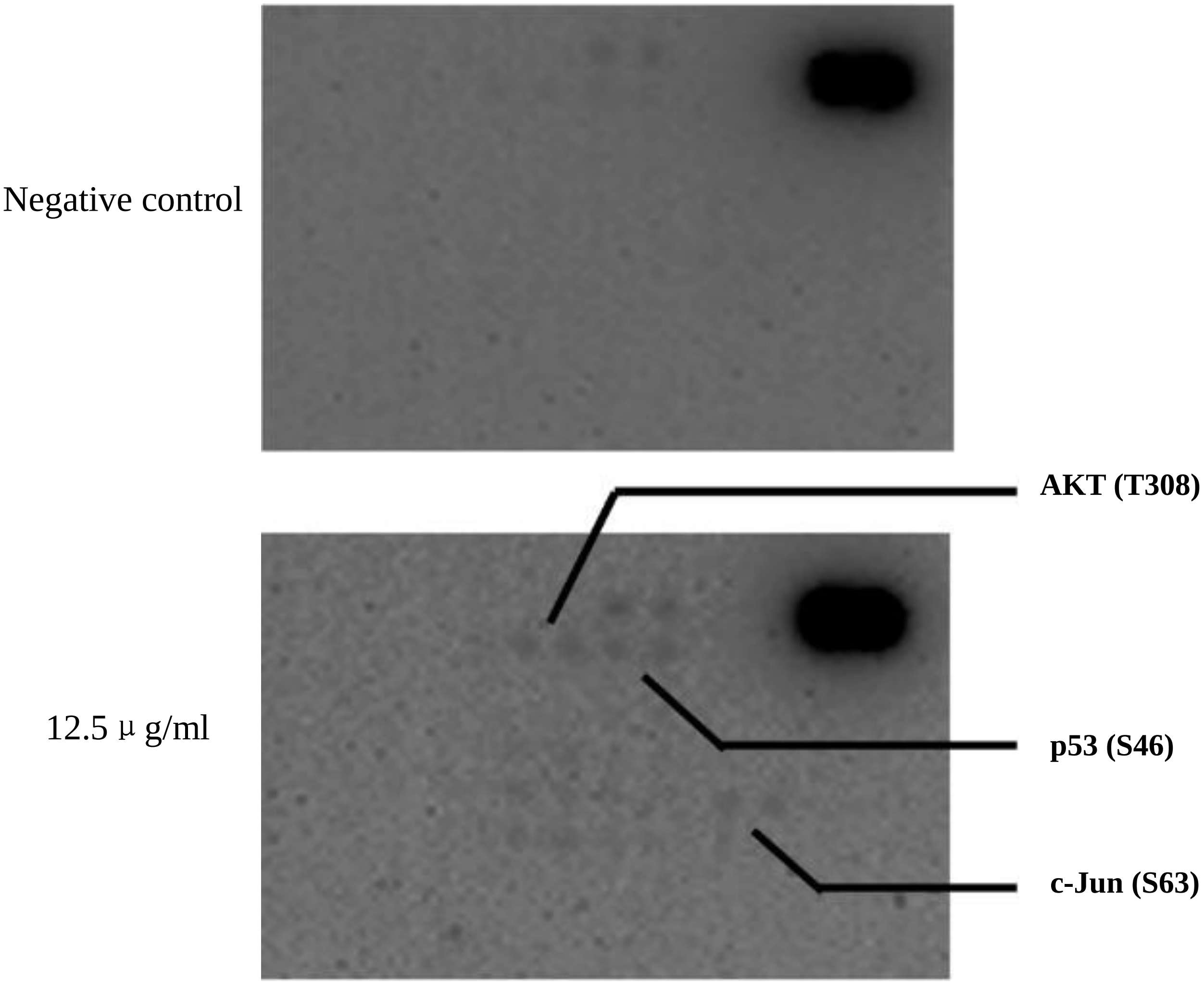

The previous non-editable figure

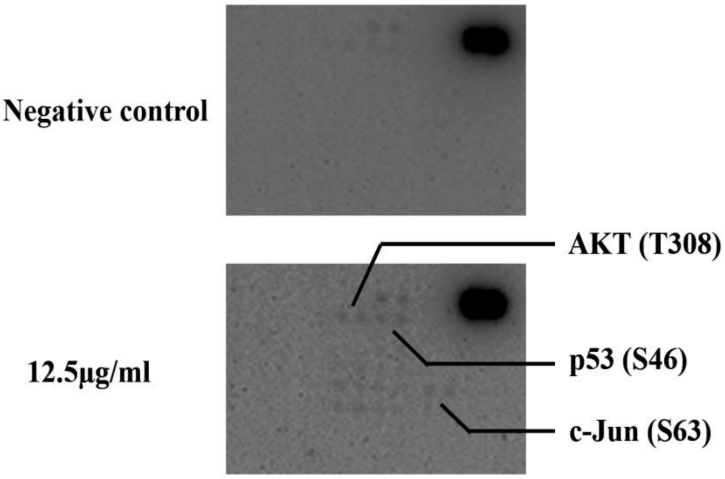

Supplement: Supplementary file 1 — Supplementary Data. Result of human phospho-kinase array assays. Allicin activated the AKT, P53, and JNK (c-Jun) pathway in SKOV3 cells were detected by human phospho-kinase array analysis. As shown, the first photo was negative control while the second was allicin (12.5μg/ml) intervention group; As a result, the signals of AKT and P53 were weakly different between the two photos; However, the signal of JNK was extremely increased in allicin group compared with the negative control. Therefore, JNK pathway was novel finding in this setting. [file 378684.f1.pdf]
